# Adherence to Iron-Folate Supplementation and Associated Factors among Pastoralist's Pregnant Women in Burji Districts, Segen Area People's Zone, Southern Ethiopia: Community-Based Cross-Sectional Study

**DOI:** 10.1155/2018/2365362

**Published:** 2018-12-31

**Authors:** Negussie Boti, Tezera Bekele, Wanzahun Godana, Eskeziyaw Getahun, Feleke Gebremeskel, Behailu Tsegaye, Bilcha Oumer

**Affiliations:** ^1^Department of Public Health, College of Medicine and Health Sciences, Arba Minch University, Arba Minch, Ethiopia; ^2^Department of Biomedical Sciences, College of Medicine and Health Sciences, Arba Minch University, Arba Minch, Ethiopia; ^3^Department of Midwifery, College of Health Sciences, Arba Minch, Ethiopia

## Abstract

**Background:**

Iron deficiency anemia among pregnant women is one of the most common public health problems in developing country particularly in Ethiopia. Iron/folic acid supplementation with optimal adherence is the main cost-effective strategy for prevention and control of iron deficiency anemia in pregnant women. However, level of adherence to iron/folic acid supplementation and its associated factors were not well identified in study area. Therefore, the aim of this study was to determine the level of adherence to iron/folic acid supplementation and associated factors among pregnant women in Burji Districts, southern Ethiopia.

**Methods:**

A community-based cross-sectional study was conducted among 317 pregnant women in Burji Districts from March to April 2017 using interviewer administered questionnaires. Data were entered into Epi Info 3.5.1 and exported to SPSS version 20.0 for analysis. Binary and multivariable logistic regression was used to identify factors associated with iron/folic acid supplementation. Adjusted odds ratio (AOR) with 95% confidence interval (CI) and p-value <0.05 were used to declare statistical significance.

**Results:**

Among women participating in the study, 163(51.4%) were adherent to iron/folate acid supplementation. Factors significantly associated with adherence to iron and folic acid supplementation were maternal educational status (AOR: 2.47, 95% CI: 1.13-4.97), early registration for ANC (AOR: 2.49, 95% CI: 1.45 – 4.27), history of anemia during current pregnancy (AOR: 2.02, 95% CI: 1.09-3.72), and knowledge about iron and folic acid supplementation (AOR: 1.96, 95% CI: 1.02-3.76). Forgetfulness and fear of side effects were among the leading reasons of pregnant women for nonadherence to iron and folic acid supplementation.

**Conclusions:**

This study revealed that adherence to iron /folic acid supplementation was found to be 51.4%. Maternal educational status, early registration for ANC, history of anemia during current pregnancy, and knowledge about iron and folic acid supplementation were significant factors associated with adherence to iron/folic acid supplementation among pastoralist's pregnant women. Therefore, anemia prevention strategy should include strengthening giving awareness, counseling, strengthening community health education, and participation in health programs which are necessary to improve the uptake of iron/folic acid supplements.

## 1. Introduction

Anemia is a global public health problem affecting two billion people worldwide [1]. The primary cause of anemia is iron deficiency, a condition caused by inadequate intake or low absorption of iron, the increased demands during pregnancy, and loss of iron through menstruation [1–3].

Pregnant women are the most prone groups for IDA [4, 5]. During pregnancy, physiological iron requirements are the highest and the amount of iron absorbed from the diet is not sufficient to meet requirements during pregnancy [6, 7]. However, plasma expansion being increased in the second trimesters, the dietary intake of the two elements cannot meet the increased need during pregnancy which leads to IDA [6]. Anemia during pregnancy leads to low birth weight, lowered resistance to infection, poor cognitive development, and reduced work capacity [3, 8, 9].

Iron and folic acid supplementation have been the preferred intervention to prevent IDA among pregnant women that may help to improve maternal and fetal outcomes because it is essential to the normal development of the spine, brain, and skull of the fetus [1, 3]. Folate also supports the pregnant woman's expanding blood volume and growing maternal and fetal tissues [2, 7].

WHO recommends that in the first four weeks of pregnancy all pregnant women should be receive a standard dose of 30–60 mg iron and 400 *μ*g folic acid beginning as soon as possible during the first trimester of pregnancy [1]. Accordingly, in Ethiopia, the national guideline for control and prevention of micronutrient deficiencies highlights the need of daily iron supplementation for at least 6 months during pregnancy and 3 months postpartum [3]. Iron-folic acid (IFA) adherence is the extent to which patients take medication or condition of sticking to dose and time for taking iron/folate supplements as prescribed by their healthcare providers or per recommendations [10, 11]. Women are said to adhere to an iron/folic acid supplement if they took 65% or more of the supplement, equivalent to taking the supplement at least 4 days a week [12]. However, oral iron-folate supplementation is hindered by different factors such as poor adherence to regimens, frequency of side effects, gastrointestinal side effects, inadequate supply of tablets, lack of counseling of pregnant women by health care providers concerning to the utilization of tablets and possible side-effects of the supplementation, poor utilization of ANC services, lack of knowledge on the IFA tablet, and lack of knowledge on anemia, but experts suggest that 1000 mg of iron is needed for mother and fetus during pregnancy [2, 13, 14].

Therefore, to control problem associated with IDA and its adverse health consequences affecting both pregnant mother and their neonate in Ethiopia especially in study area provision iron and folic acid supplementation is one of strategies to all pregnant during pregnancy. Therefore identifying the level of adherence and its determinants in high-risk groups, such as pregnant women, would be essential for evidence-based intervention. There is no updated data about the level of Adherence to IFAS among pregnant women in the study area. Therefore, the aim of this study is to determine the level of adherence and associated factors among pregnant women in Burji Districts, Southern Ethiopia.

## 2. Methods and Materials

### 2.1. Study Area and Design

This study was conducted in Burji District. Burji District is one of the five districts in Segen Area Peoples Zone of the Southern Nations, Nationalities, and Peoples Region (SNNPR) of Ethiopia. The administrative center of Burji is Soyama. Burji District has two urban and twenty-four rural kebeles. There are five health centers and twenty-four health posts, which provide routine antenatal care services to the community. Based on the 2007 Census conducted by the Central Statistical Agency of Ethiopia (CSA), this woreda has a total population of 55,681, of whom 27,207 are men and 28,474 women [15]. A community-based cross-sectional study design was conducted from March to April 2017.

### 2.2. Source and Study Populations

All pregnant women aged 15–49 years who were living in the district were the source population. The study population was pregnant women aged 15–49 years who were attending ANC and being supplied with the iron-folate supplement at least for a month during the data collection period. Pregnant women who were severely ill, those who had a mental illness, and who were living in the study area less than six months were excluded.

### 2.3. Sample Size Determination

The sample size was calculated using the single population approach and it was calculated using Epi Info version 7.02 statistical software package with the assumption of 95% confidence level (Z*α*/2= 1.96), 80% power (Z*β*= 0.84), and P =74.9% on the proportion of adherence to iron-folic acid supplementation among the pregnant women, which was taken from a previous study conducted rural districts of Ethiopia [16]. Considering 10% nonresponse rate, the maximum sample size was 317 study participants.

### 2.4. Sampling Procedure

Simple random sampling technique was used to select study participants using average previous month's antenatal care users used as the sampling frame which is obtained from a community family folder. In Burji District, there were twenty-six kebeles; among those, eight kebeles were selected using a lottery method. A proportional to size allocation was employed to obtain the sample size for selected kebeles. Prior to the actual data collection being given, the list of study subjects was identified by using community health management information system (CHMIS) folder. Finally, the study participants were selected by using a simple random sampling method. If in case more than one eligible respondent is found in the household, only one respondent had been chosen by lottery method

### 2.5. Operational Definition

#### 2.5.1. Adherence

Pregnant mothers took at least 65% of the expected dose of the iron-folate tablets in the previous week before the study, which is equivalent to consuming at least 1 tablet daily for 4 days in the week consecutively or consuming 20 tablets in a month daily without missing the prescribed doses [1, 17].

#### 2.5.2. Knowledge of Iron-Folate Supplement

It was measured by summing up 8 multiple-choice items. A correct answer was given one mark, while a wrong answer was not given any mark. If the pregnant women scored median and above, then they have good knowledge and those pregnant women who scored below the median, they have poor knowledge [18].

### 2.6. Data Collection Procedure and Data Quality Control

Data were collected using structured questionnaire adapted from different peer-reviewed published works of literature [4, 17, 19]. A structured pretested interviewer administered questionnaire was developed in English and then translated into Amharic language for simplicity and then backtranslated to English language for its consistency by two different language expert individuals who speak both English and Amharic fluently. The questionnaire has four parts sociodemographics, obstetrics characteristics, health service-related characteristics, and client related characteristics which were used. Pretesting of the questionnaire was done on 5% of the sample at Arba Minch Zuriha district who was not included in the study; that was a week before commencement of the actual data collection. Based on the pretest, a questionnaire was corrected to ensure clarity, wording, and logic sequence and skip patterns. The data was collected by nine diploma health professionals and supervised by four trained health professionals who had BSc. Data collectors and supervisor were trained for one day on objectives of the study, how to keep confidentiality of information, the contents of the questionnaire, filling data collection format, and data quality management by the principal investigator. At the end of each day, questionnaires were reviewed and cross-checked for completeness, accuracy, and consistency by the supervisor and principal investigator and corrective measures were taken. The overall activity was supervised by the principal investigator of the study.

### 2.7. Data Processing and Analysis

The collected data were coded, cleaned, and entered by Epi-Info version 7.2 and exported to statistical package for social science (SPSS) version 20.0 for analysis. Descriptive statistics including tables and proportion were used to describe the data. Binary and a multivariable logistic regression analyses were performed to see the association between dependent and independent variables. Variables that showed association in binary logistic regression analysis and which have P-value less than 0.20 were entered into multivariable logistic regression analysis model. Finally multivariable logistic regression model is used for controlling confounding factors and to identify significant factors associated with dependent variable. An effort was made to assess whether the necessary assumptions for the application of multivariable logistic regression analysis were fulfilled. In this regard, the Hosmer and Lemeshow's goodness-of-fit test with large p-value (p>0.05) was checked to see good fitness. Multicollinearity and confounding effect was checked by using standard error. The variable without multicollinearity was entered into multivariable model. At the end AOR with 95% CI, P-value <0.05 was considered statistically significant.

### 2.8. Ethical Consideration

The study was conducted after getting ethical clearance from Arba Minch University, College of Medicine and Health Science Institutional Review Board (IRB). Support letter was obtained from Zonal Health Department as per the recommendation letter from the public health department. Verbal consent was secured from study participants after explaining the objective and purpose of the study to each study participant. The participants were also assured about the confidentiality.

## 3. Results

### 3.1. Socioeconomic and Demographic Characteristics of Pregnant Women

A total of 317 pregnant women whose age ranges from 18 to 49 years were interviewed. The mean age was found to be 25.6 with (±SD) 4.9 years and the majority of pregnant women, 270 (85.2%), were age group from 20 to 34 years. Two hundred thirty-eight (75.1%) of study participants have resided in the urban area. The majority, 248 (78.2%), was married. The majority, 188 (89.3%), of pregnant women had no formal education while 47 (14.8%) pregnant women had primary educational status, but the majority of husbands, 129 (40.7%), had primary educational status. About 292 (92.1%) mothers were housewives and 169 (53.3%) mothers had more than six children (Table 1).

### 3.2. Obstetric Related Characteristics of Pregnant Women

One hundred eighty (56.8%) and 178 (56.1%) were primigravidae and primiparous, while 137 (43.2%) and 95 (30%) were multigravida and multiparous. About 185 (58.4%) mothers visited the ANC clinic within the last 16 weeks of gestation. The majority of pregnant women, 217 (68.5%), visited ANC for follow-up more than four times in health institution. About 32 (10.1%) respondents had the history of abortion; also 12 (3.8%) respondents had the history of stillbirth. On current pregnancy, 141 (44.5%) pregnant women had the history of anemia (Table 2).

### 3.3. Knowledge of Pregnant Women about Anemia and IFAS

Accordingly, 170 (53.6%) respondents had good knowledge of anemia (scored median and above) and 147 (46.4%) respondents had poor knowledge of anemia (scored below the median). Out of all study participants, 148 (46.7%) respondents had good knowledge of IFAS (scored median and above) and 169 (53.3%) respondents had poor knowledge of IFAS (scored below the median).

### 3.4. Adherence to the Iron/Folate Supplementation Based on Pill Counting

**Figure 1:**
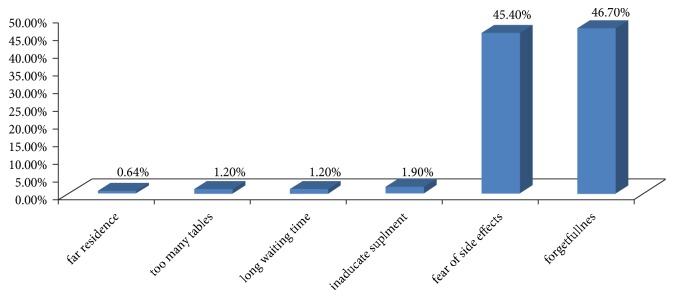
Reason for not adherence among Pastoralist's pregnant women in Burji Districts, Segen Area Zone of Southern Nations, Nationalities and Peoples Region, Ethiopia, March–April 2017.

Adherence to the iron/folate supplementation was measured based on pill counting [9]. The finding of this study reveals that one hundred sixty-three (51.4%) of pregnant women have adhered to IFAS (took ≥4 tablets per week or consuming 20 tablets in a month daily without missing the prescribed doses) and 154 (48.6%) did not adhere to IFAS (took <4 tablets per week or consuming table with missing the prescribed doses). The reasons for adherence were getting counseling from health extension worker (245, 86.5%), followed by fear of illness if not taking the supplement (106, 42.3%) and getting family support (43, 15.2%). The leading reason for not adhering was forgetfulness to take table, 135 (46.7%), then taking too many pills, 72(22.7%), followed by fear of side effects, 70 (45.4%). From those who express side effects, the commonest side effect was heartburn, 153 (48.3%), and vomiting, 137 (43.2 %) (Figure 1).

### 3.5. Factors Associated with Adherence to IFAS among Pregnant Women

The multivariable logistic regression showed that maternal educational status, time of ANC registration, knowledge of anemia, and knowledge of IFAS were statistically significant with adherence of IFAS. The pregnant women who had secondary and above education were nearly 2.5 times (AOR=2.47, 95% CI=1.13-4.97) more likely to have adhered to IFAS than those pregnant women who had primary education. The pregnant women who had early registration (≤16 weeks) for ANC were 2.49 times (AOR= 2.49, 95% CI=1.45 – 4.27 more likely to adhere to IFAS than those who had late registration (>16 weeks). The pregnant women who had the history of anemia during current pregnancy were 2 times (AOR= 2.02, 95% CI= 1.09-3.72) more likely to adhere to IFAS than those who did not have any symptom of illness. The pregnant women who had good knowledge of IFAS were nearly 2 times (AOR= 1.96, 95% CI= 1.02-3.76) more likely to have adhered to IFAS than those who had poor knowledge of IFAS (Table 3).

## 4. Discussion

Pregnant women are among the most vulnerable groups of iron deficiency anemia. The World Health Organization recommends giving all pregnant women a standard dose of 60mg iron and 400*μ*g folic acid daily for 6-month duration. Iron and folic acid supplementation are among the feasible ways to prevent anemia during pregnancy. However, maternal adherence to iron and folic acid supplementation plays a major role in the prevention and treatment of iron deficiency anemia. Thus, the aim of this study was to determine the adherence status and identify factors associated with iron and folic acid supplementation among pregnant women who live in a study area.

Among women participating in the study, 163 (51.4%) were adherent to iron/folate acid supplementation. This finding was higher than the finding of study conducted in Mecha district, Northwest Ethiopia (20.4%) [19], the one conducted in North Western Zone of Tigray, Ethiopia (39.2%) [17], and the study conducted in Kenya (24.5%) [14]. This might be associated with increased knowledge of pregnant women about anemia and IFA supplementation (through medical advice and media).

In addition, the result of adherence found in this study was lower than result found in eight rural districts in SNNP, Ethiopia, 2014, which was 74.9% average level of adherence rate of pregnant women in the area [16] and the study was done in South India (64.7%) [20]. This inconsistency may be due to cultural, geographical location, and availability of drugs in the health center. Another might be that this study was done in rural-based area and the pregnant women may not get information from the health center and women in rural areas were probably less educated than those in urban areas which could have contributed to the lower level than the regional level. Also, there might be a misunderstanding of the need to take the tablets throughout pregnancy due to inadequate counseling and beliefs against consuming medications during pregnancy; that is, the medications may cause too much blood or a big baby, making delivery more difficult.

This study revealed that maternal education status had a significant association with adherence to iron and folic acid supplementation. Pregnant women who had secondary and above education were nearly 2.5 times more likely to adhere to IFAS than those pregnant women who had primary education. The finding is supported by other studies done in Mecha district, Northwest Ethiopia, Nepal, and Indonesia [18, 21, 22].

This might be associated with educated women who are likely to have better knowledge and access to information about iron deficiency anemia and therapy, the benefits of supplements, and pregnancy in general. Secondly, it might be due to the fact that education would increase the women's access to information through reading and understanding the benefit of the supplement. Third, it might be associated with the notion that education is more likely to enhance female awareness of micronutrient deficiency and ways to overcome these deficiencies. And it might be associated with the fact that educated women have greater ability to stick to health care inputs such as IFA which offer better care for both the infant and the mother.

The present study reveals that adherence was better observed among pregnant women who were early registered for antenatal care service as compared to late registered women. Accordingly, pregnant women who had early registration (≤16 weeks) for ANC were 2.49 times more likely to have adhered to IFAS than those who had late registration (>16 weeks). The result of this study is supported by other studies done in Tigray, Ethiopia, Indonesia, and India [17, 20, 21]. The reason might be that pregnant women who had early registration for ANC services probably had better concern for their pregnancy and had more ANC visits which in turn leads to getting better medical advice and ultimately increased knowledge about anemia and iron and folic acid supplementation.

Similarly, a history of anemia during current pregnancy was associated with greater adherence to iron supplementation. Pregnant women who had a history of anemia during current pregnancy were 2.02 times more likely to have adhered to IFAS than those who did not have any symptom of illness. This finding was supported by study conducted in Tigray, Ethiopia, and study conducted in Mecha district, Western Amhara [17, 19]. This might be due to relief from symptoms and fear of further complications and the provider may give more emphasis to anemic clients on counseling compared to non-academic clients. Secondary it might be the fact that the number of iron/folic acid pills that should be taken according to the prescription will entirely depend on the frequency of illness that the mother had. If the mother did not have any symptom of illness, she would not take the amount of pills she should take.

Another factor that had a significant association with iron and folic acid supplementation adherence in this study was knowledge of iron and folic acid supplementation. Pregnant women who had good knowledge of IFAS were nearly 2 times more likely to adhere to IFAS than those who had poor knowledge of IFAS. This finding is supported by other studies done in eight rural districts of Ethiopia and India [16, 20]. The possible reason is that those pregnant women who had good knowledge of iron and folic acid supplementation were aware of the tablets importance, side effect, how it is taken, and complication if missed. Secondly, this might be associated with the fact that knowledge helps women to have a good perception of the benefits of taking iron tablets.

Forgetfulness and fear of side effect were the leading reason of pregnant women for nonadherence to iron and folic acid supplementation. The finding of this study is supported by other studies done in Ethiopia [4–6]. A possible explanation for forgetfulness was because most pregnant women in a rural part of Ethiopia were tired at night time because they spent the daytime with different activities. The majority of women have a misunderstanding about taking the tablets due to inadequate counseling because most of the community in rural part believes that consuming medications during pregnancy may make delivery more difficult. So better counseling might decrease the high occurrence of side effects by increasing the psychological tolerance of women to side effects of the tablet.

### 4.1. Strength and Limitations of the Study

The possible strength of this study was using a gold standard method of measuring the number of iron/folic acid uptake pills; counting method was used which is the best predictor of adherence rate. Limitation of this might be cross-sectional in nature or might be chicken egg dilemma and it does not test the hypothesis.

## 5. Conclusions

The finding of this study revealed that the adherence rate was 51.4%. Maternal educational status, early registration for antenatal care services, history of anemia during current pregnancy, and knowledge of pregnant women about iron and folic acid supplementation were significantly associated with pregnant women adherence to iron and folic acid supplementation. Forgetfulness and fear of side effects were among the leading reasons of pregnant women for nonadherence to iron and folic acid supplementation. Therefore, health professionals and responsible bodies should give attention to advising and counseling a pregnant woman on benefits and starting time of iron folate supplementation.

## Figures and Tables

**Table 1:** Sociodemographic and economic characteristics of Pastoralist's pregnant mothers in Burji Districts, Segen Area Zone of Southern Nations, Nationalities and Peoples Region, Ethiopia, March-April 2017 (N=317).

**Variables**	**Categories**	**Frequency(n)**	**Percent (**%**)**
**Age in years**	15-19	22	6.9
	20-34	270	85.2
	≥35	25	7.9

**Residence **	Rural	79	24.9
	Urban	238	75.1

**Marital status**	Single	24	7.6
	Married	248	78.2
	Divorced	34	10.7
	Widowed	11	3.5

**Maternal educational status **	Have no formal education	188	89.3
	Primary	47	14.8
	Secondary and above	82	28.8

**Husband educational status**	Have no formal education	106	33.4
	Primary	129	40.7
	Secondary and above	82	25.9

**Maternal Occupational status**	Housewife/Farmer	292	92.1
	Merchants	12	3.8
	Employers	12	3.8
	Others	1	0.3

**Number of lived children **	1-3	45	14.2
	4-6	103	32.5
	>6	169	53.3

**Table 2:** Obstetric related characteristics of Pastoralist's Pregnant mothers in Burji Districts, Segen Area Zone of Southern Nations, Nationalities and Peoples Region, Ethiopia, March-April 2017 (N=317).

**Variables**	**Categories**	**Frequency(n)**	**Percent (**%**)**
**Number of pregnancy**	Primigravida	180	56.8
	Multigravida	137	43.2

**Number of deliveries**	Nulliparous	44	13.9
	Primiparous	178	56.1
	Multiparous	95	30

**Number of Visits**	≤4	217	68.5
	>4	100	31.5

**Gestational age at the start of ANC**	First trimester	121	38.2
	Second trimester	177	55.8
	Third trimester	19	6

**Time of registration **	<16weeks (Early)	185	58.4
	≥16weeks (Late)	132	41.6

**History of Abortion**	Yes	32	10.1
	No	285	89.9

**History of still birth **	Yes	12	3.8
	No	305	96.2

**History of Anemia on current pregnancy **	Yes	141	44.5
	No	176	55.5

**Table 3:** Factors associated with adherence to IFAS among Pastoralist's Pregnant women in Burji Districts, Segen Area Zone of Southern Nations, Nationalities and Peoples Region, Ethiopia, March-April 2017(N=317).

**Variables**	**Categories**	**Adherence status**		**COR(95**%** CI)**	**AOR(95**%** CI)**
		**Adhered**	**Not Adhered**		
		**N (**%**)**	**N (**%**)**		
**Age**	15-19	11(6.7)	11(7.1)	0.67(0.21,2.12)	0.83(0.27,2.53)
	20-34	142(87.1)	128(83.1)	0.60(0.26,1.39)*∗*	1.24(0.29,5.23)
	≥35	10(6.1)	15(9.7)	1	**1**

**Maternal educational status**	Have No formal education	82(50.3)	106(68.8)	1	**1**
	Primary	31(19	16(10.4)	2.02 (1.19,3.43)*∗*	0.77(0.34,1.71)
	Secondary and above	50(30.7)	32(20.8)	0.81 (0.38,1.71)	**2.47(1.13,4.97)** **∗** **∗**

**Husband educational status**	Have No formal education	44(27)	62(40.2)	1	1
	Primary	69(42.3)	60(39)	2.20 (1.22,3.97)*∗*	1.46(0.72,2.94)
	Secondary and above	50(30.7)	32(20.8)	1.36(0.77,2.39)	2.10(0.92,4.39)

**Maternal occupational status**	Housewife	109(66.9)	113(73.4)	1	**1**
	Merchants	25(15.3)	25(16.2)	1.81(0.79,4.13)	1.98(0.82,4.79)
	Employers	29(17.8)	16(10.4)	1.88(0.97,3.65)*∗*	2.13(0.94,4.37)

**Family size**	1-3	25(15.3)	20(13)	1	1
	4-6	54(33.1)	49(31.8)	1.13(0.56,2.29)	1.43(0.61,3.36)
	>6	84(51.5)	85(55.2)	1.27(0.65,2.45)	1.05(0.38,2.88)

**Gravidity**	Primigravida	98(60.1)	82(53.2)	1	1
	Multigravida	65(39.9)	72(46.8)	1.32(0.85, 2.07)*∗*	1.17(0.52,2.61)

**Trimester**	First	58(35.6)	63(40.9)	1	1
	Second	98(60.1)	79(51.3)	0.74 (0.47,1.18)*∗*	1.19(0.34,4.17)
	Third	7(4.3)	12(7.8)	1.58(0.58,4.28)	0.51(0.71,1.47)

**Time of Registration**	Early(≤16weeks)	100(61.3)	85(55.2)	1.29(0.82,2.02)*∗*	**2.49(1.45,4.27)** **∗** **∗**
	Late(>16weeks)	63(38.7)	69(44.8)	1	**1**

**History of Anemia **	Yes	74(45.4)	67(43.5)	1.08(0.69,1.68)	**2.02(1.09,3.72)** **∗** **∗**
	No	89(54.6)	87(56.5)	1	1

**Knowledge of Anemia**	Good	84(51.5)	86(55.8)	1.19(0.76,1.85)	1.21(0.75,1.95)
	Poor	79(48.5)	68(44.2)	1	1

**Knowledge of IFAS**	Good	52(31.9)	27(17.5)	2.2(1.29,3.74)*∗*	**1.96(1.02,3.76**)*∗∗*
	Poor	111(68.1)	127(82.5)	1	1

AOR = adjusted odd ratio; CI = confidence interval; COR = crude odd ratio; *∗∗* = statistically significant

## Data Availability

The data used to support the findings of this study are available from the corresponding author upon request.

## Abbreviations

ANC: Antenatal care
AOR: Adjusted odds ratio
CI: Confidence interval
COR: Crude odds ratio
IDA: Iodine deficiency anemia
IFA: Iron folic acid
IFAS: Iron folic acid supplementation
OR: Odds ratio
SNNPR: Southern Nations Nationalities Peoples Region
SPSS: Statistical Package for Social Science
WHO: World Health Organization.
